# How do care-provider and home exercise program characteristics affect patient adherence in chronic neck and back pain: a qualitative study

**DOI:** 10.1186/1472-6963-10-60

**Published:** 2010-03-10

**Authors:** Pilar Escolar-Reina, Francesc Medina-Mirapeix, Juan J Gascón-Cánovas, Joaquina Montilla-Herrador, Francisco J Jimeno-Serrano, Silvana L de Oliveira Sousa, M Elena del Baño-Aledo, Rafael Lomas-Vega

**Affiliations:** 1 Department of Physical Therapy, University of Murcia, Spain; 2 Department of Public Health and Preventive Medicine, University of Murcia, Spain; 3 Department of Physical Therapy, Central Unit of Anatomy, Catholic University San Antonio of Murcia, Spain; 4 Department of Health Sciences, University of Jaén, Spain

## Abstract

**Background:**

The aim of this study is to explore perceptions of people with chronic neck or low back pain about how characteristics of home exercise programs and care-provider style during clinical encounters may affect adherence to exercises.

**Methods:**

This is a qualitative study consisting of seven focus groups, with a total of 34 participants presenting chronic neck or low back pain. The subjects were included if they were receiving physiotherapy treatment and were prescribed home-based exercises.

**Results:**

Two themes emerged: home-based exercise programme conditions and care provider's style. In the first theme, the participants described their positive and negative experiences regarding time consumption, complexity and effects of prescribed exercises. In the second theme, participants perceived more bonding to prescribed exercises when their care provider presented knowledge about the disease, promoted feedback and motivation during exercise instruction, gave them reminders to exercise, or monitored their results and adherence to exercises.

**Conclusions:**

Our experiential findings indicate that patient's adherence to home-based exercise is more likely to happen when care providers' style and the content of exercise programme are positively experienced. These findings provide additional information to health care providers, by showing which issues should be considered when delivering health care to patients presenting chronic neck or back pain.

## Background

Neck and low back pain are prevalent and they are the major cause of work disability, being responsible for high costs to society [1,2]. Recurrence of neck and low back pain are common and their course is variable [3-5], with 10-15% of cases leading to chronic pain [6,7]. Exercise therapy commonly forms part of the treatment prescribed by care providers to patients presenting low back or neck pain. Systematic reviews have concluded that exercise appears to be effective in decreasing pain and improving function [8-11]. Exercises are often instructed individually and prescribed to be performed at home [12]. Although home-based exercises vary greatly in the method of delivery and content [7,13,14], different programmes appear to have similar effects on patients [15,16].

Scientific evidence suggests that inadequate adherence to home-based exercises may attenuate the treatment's efficacy [10,17,18]. It has also been proposed that many recurrent cases of low back pain could have been avoided if patients had adhered to their home programs [19,20]. Nevertheless, several studies reported that adherence to exercise is often a serious issue for patients with neck or low back pain. Differences in the definition of adherence used, measurement and estimative of how many patients do not comply with their prescribed exercises vary, but evidence converge on a figure of 50% or higher [17,18,21,22].

Research suggests that certain conducts of care-provider, such as giving patients positive incentives, giving feedback about their progress and treatment, or monitoring their exercise performance, they all influence in the adherence to home exercise programs [9,23-25]. In addition, other studies also evidenced that patients usually experience some intrinsic factors which are understood to bring difficulties in the performance of home-based exercises. The most common factors are the lack of time to exercise, and the inability to fit the exercises into their daily routine [26].

Most of those studies investigating the influences of patients' adherence employed highly structured questionnaires intended to obtain responses to questions that the researchers had previously identified to be relevant[27]. Although a few qualitative studies have studied these factors from the perspective of lower back pain patients [28,29], no study to date have explored the factors related to patient-provider issues. Despite the fact that previous studies explored the role of care provider's conduct and the content of home-based exercise programs towards the patients' compliance with prescribed exercises, further investigation is needed to understand which aspects of home-based programmes and clinical settings may increase adherence to prescribed exercise in a low back or neck pain patient population. This issue, explored from the patients' perspective, is important due to the fact that many patients exercising because of chronic pain usually make active decisions about their own exercises, rather than being simply passive recipients of health care [30].

The aim of this study was to explore how the intrinsic characteristics of home-based exercise programme or care provider' style in clinical settings affects chronic neck or low back pain patients' adherence to prescribed exercise.

## Methods

### Study design

The qualitative focus group design was selected due to the fact that group interactions provide means of obtaining rich and detailed data from subjects who participated in home-based exercise programmes [23].

### Participants

Inclusion criteria for the study were: patients 18 years of age or older, who could speak, read and understand Spanish. They should have had at least one episode of mechanical chronic neck or low back pain at least eight weeks prior attending physiotherapy treatment, had attended physiotherapy treatment in the last 3 months, and had participated in a home-based exercise program. Neck pain was defined as a pain located in the area limited between the occipital and the third thoracic vertebra [31]. Likewise, back pain was defined as pain perceived below the shoulder blades, above the gluteus fold, with or without lower limbs referred symptoms [2]. Exclusion criteria were: patients presenting mechanical chronic neck or low back pain due to trauma, or patients presenting inability to participate in focus groups due to physical or mental disability (i.e. deafness, blindness, or learning disability).

### Recruitment

The study was approved by the Committee of Ethic and Research of University of Murcia. Recruitment was made by inviting patients from four public primary health care centres in the region of Murcia, Spain. These centres were selected because patients presenting mechanical neck or low back pain are often attended by both clinical appointments and prescribed home-based exercises, during the period of treatment and follow-up period.

Following the Committee of Ethic and Research approval, the eligible patients were identified in each health care centre by consultation of patient records. We initially extracted the subjects with neck or low back pain from a list of patients referred to physiotherapy treatment. The list contained relevant data, such as name, diagnosis and date. Afterwards, the initial selection was analysed by the in-house physiotherapist using the inclusion/exclusion criteria. In total, 94 were eligible participants.

Purposive sampling strategy [32] was used to include subjects with different age, gender, and clinical conditions. This allowed for the selection of participants who could best provide insight into specific and personal experiences regarding the issues being examined, rather than obtaining a representative sample, as would be sought in quantitative research. Although we were aware that the final sample size was dependent on the saturation of information, we initially selected forty-two subjects.

The first contact with each patient was made by an invitation letter, and later they were contacted by two telephone calls. The letter contained an explanatory statement, date, and place of meeting. The letter was not signed by any care provider and the groups were not interviewed in the health centre, but in public and neutral locations (i.e. city hall) instead, in order to ensure that the subjects were not intimidated to participate. In the first phone call, people were asked questions to screening of inclusion/exclusion criteria, and to check their willingness to participate. When several patients declined to participate, new patients presenting similar characteristics were invited to ascertain a group with a minimum size of 4 members. In the second phone call, subjects of each focus group were reminded 2-3 days prior interview to confirm their presence.

Homogeneous and heterogeneous criteria were used to form the groups. On the one hand, participants had consistency in gender, in order to avoid apprehension in discussing health issues in the presence of the opposite gender. On the other hand, we tried to form heterogeneous groups by age and clinical condition (neck/back pain) with the intention to add variability of experiences with the aim to stimulate discussions.

### Data collection

Two researchers conducted the discussions, one moderator with a PhD degree and experience in focus groups, and one assistant. A topic guide containing pre-determined questions was used (Appendix). This guide was initially formed from a literature review as previously described [33] and varied slightly from the initial interview's agenda. Additional questions were included according themes started to emerge from the initial focus groups [34]. An audiotape was used for data collection during the interviews, and a videotape and field notes were used to record the subjects' non-verbal language or incomplete or sarcastic expressions. Patients were reassured of terms of confidentiality before the beginning of each interview session and were given the right to consent by a consent form. Every subject participating in the focus groups accepted to be interviewed before the session begun. Seven focus groups were formed because emergent themes were consolidated after these seven groups [34]. Focus groups sizes varied from four to six participants, and the sessions lasted from 40 to 80 minutes.

The sessions were literally transcribed by an independent assistant. Each participant was assigned a code number for data entry and quotations. Notes taken during the interviews, and the moderator's reflections were used to write a report of each interview.

### Data analysis

The principles of Grounded Theory [34,35] were used in the analysis process in order to originate a theoretical proposal grounded in the participants' views [34]. The following steps were used: a first reading of all transcripts to get an overall impression of content; segmentation of the transcripts sentences or paragraphs and codification of phrases; generation of themes or categories; and identification of any relationships among themes or categories [36].

Three authors (PER, FMM, JJGC) independently segmented the phrases, labelled them into categories, and combined the categories into key themes. The authors reviewed and compared their findings in order to form an agreement on themes and categories, before the identification of combination proceeded. Three rounds of coding and discussion took place with the intention of enhancing credibility of the analysis used, and to develop clearer themes and categories. This process was iterative with data collection, allowing new categories to be inserted, and exclusion of repetitive themes or categories from the data of subsequent group transcripts. No new themes or categories emerged at the end of the seventh focus group, which implied that the saturation was reached. To check consistency of the final emergent themes and categories, two researchers cross-checked their agreement through a blind review using codes for the same passages of 2 transcripts [37]. Any disagreements between the two researchers were resolved by discussion. Finally, PER, FMM, JJGC interconnected the themes and categories and combined them to form a theoretical model. At every step, an independent researcher (JMH) played a role of reviewer to verify if the analysis was systematically supported by the data with the intention of enhancing dependability [34]. Confirmability was enhanced when the same themes emerged from the data of subsequent groups transcripts.

## Results

**Figure 1:**
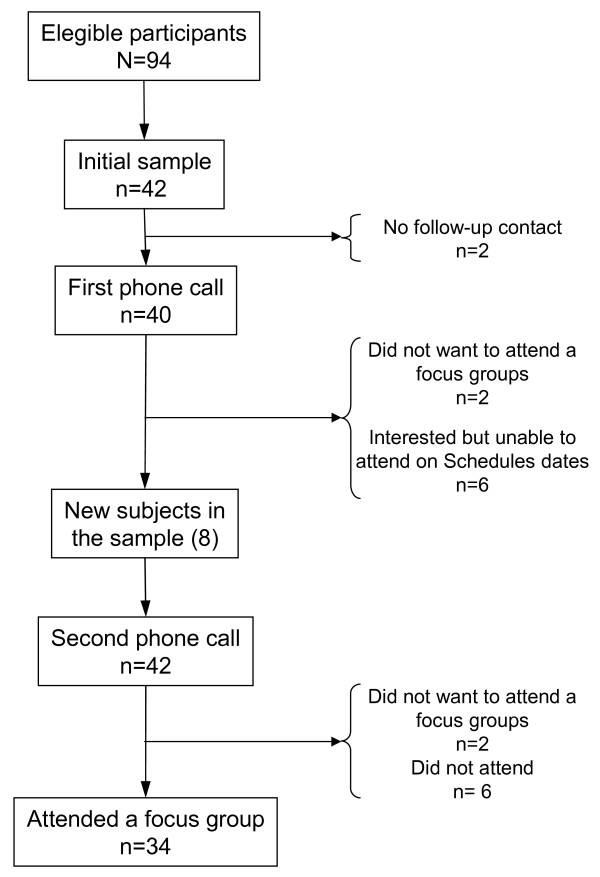
**Stages of selection process for focus group**.

Forty-two subjects were selected from an initial sample of 94 eligible participants. Initially, 8 patients were excluded either because they were unavailable to be contacted by phone, or they did not accept to attend the focus groups, or they were unable to attend the interview on scheduled dates. Consequently, 8 new patients were added to the initial sample. Initially, 40 patients were interested to attend the meeting in the second phone call, but not all of them participated in the focus groups. The progress of the stages of selection for the focus groups is illustrated in Figure 1.

In the end, there were 34 participants in this study (gender: 23 F/11 M) and 22 presented chronic neck pain. The mean age was 48 years old, and age ranged from 25 to 70 years old. All participants were receiving home-based exercise programmes. Most participants expressed their perceptions in regards to the problems they encountered to comply with the exercise programme.

**Figure 2:**
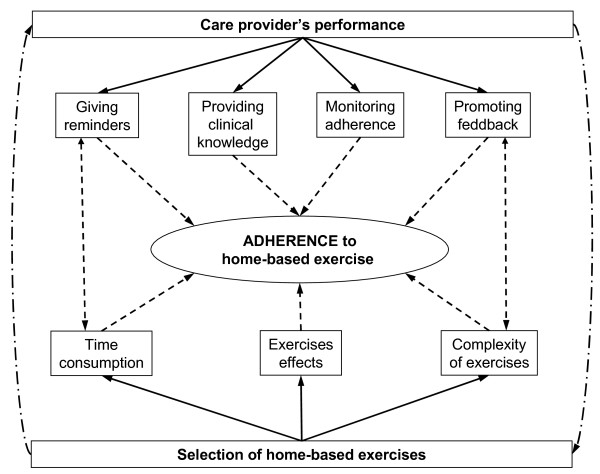
**Factors related to participant's adherence to home-based exercise programmes**.

The focus groups' results indicate that the participants interviewed in this study mentioned that the some characteristics of their care provider's performance during the period of treatment in the health care centre affected, in a way, their adherence to the home exercise program they were receiving. Participants also reported that some specific characteristics of the prescribed exercises also affected their adherence (Figure 2). The results are presented in each of the following emergent themes: (1) conditions of prescribed home-based exercise program; and (2) care provider's style. They will be presented in sub-themes with example quotes. The identification code and demographic characteristics are given for each quote below.

### Home-based exercise programme conditions

This theme comprises the following sub-themes: time consumption of home-based programme, complexity and effects of exercises. All participants reported that their experience had enabled them to identify several exercises styles, in relation to time consumption and complexity, which were problematic for their compliance to the programme. Moreover, they considered that the effects perceived during or after exercising were relevant to their adherence to the programme.

#### Time consumption of home-based programme

All participants alleged that the prescribed programmes usually require commitment in terms of time, and the need to incorporate the programme into daily routine. Consequently, participants reported that adherence to the programme was difficult when they had to spend too much time doing the exercises at home. Ten participants recognised that if the home-based programme requires a lot of time, they tend to prefer drugs by pragmatic reflection.

Participant 10: "The medication takes just one minute, while the exercises take 30 minute. Although medications may be bad for my health, they are easier to take" (Male, 55 years)

#### Complexity of exercises

High levels of complexity of prescribed exercises, difficulty in initiating the exercises, and the potential of discomfort during or after exercising were reported as the factors which impede their adherence to the exercise programme. Eight participants reported that engagement to exercise series was more difficult when specific postures or equipment preparation was required. Participants recognised that the prescribed exercises which were easy to begin were helpful for compliance with the programme. Six participants felt discomfort at home while doing the exercises prescribed in the health care centre. They recognised that those types of home exercises should have considered the differences of equipment and environment.

Participant 6: *"I didn't always do the exercises. When I only had to sit down and do the exercises it was more comfortable and easier" (Male, 48 years)*

Participant 18: *"Sometimes I do exercise, but other times I don't, because it is not as comfortable in my house as in the clinic" (Male, 58 years)*

#### Exercises effects

All participants expressed their own opinions about the relevance of positive and negative outcomes of exercises on the adherence to the whole programme. Although 7 participants reported having adverse effects during or after exercising, such as pain or swelling, 17 participants reported positive outcomes, such as improvement of impairments or quality of life. According to the participants' opinions and experiences, beneficial and adverse effects had an opposite sense on the adherence to exercises. On the one hand, the perception of adverse effects impacted negatively on adherence.

Participant 22: *"I had to stop using the bicycle because my knee was getting swollen. She also recommended that should walk, but I cannot do that either" (Male, 65 years)*

In contrast, the perceived benefits of home exercises impacted positively on the participant's adherence to the programme. However, the influences were inconsistent. The adherence to exercises increased when participants perceived its benefits, and decreased when pain was absent.

Participant 11: *"I used to do the exercises at home because then I could better move my arm. I did them for a long time, until I realized that my arm was not aching and my hand was no longer numb. Since then, I have not done the exercises" (Female, 49 years)*

### Care provider's style

Some care provider's styles were perceived to play positive or negative influence on how the participants start or continue to perform the prescribed home-based exercises. This theme brought up the following sub-themes: providing clinical knowledge, promoting feedback during exercise instruction, giving reminders, and monitoring results and adherence to exercises.

#### Providing clinical knowledge

All participants reported that the lack of clinical knowledge about the disease or goals of exercises proved to be a barrier to prescription adherence. They felt more motivated to comply with the prescription when they received explanation about their clinical condition and the treatment's justification was accurate, understandable, and convincing.

Participant 26: *"When I went to the clinic and asked the professional what I had, he explained it clearly, so I truly participated in the treatment". (Female, 43 years)*

Participant 4: *"She (professional) told me that I would get worse... and she convinced me because she explained why". (Female, 56 years)*

#### Promoting feedback during exercise instruction

Nine participants reported that adherence to exercises was difficult when their care providers failed to observe their performance while exercising in the centre. Likewise, lack of feedback and monitoring with corrections during the time the exercises were being prescribed were also reported to be negative factors to compliance with exercise prescription. According to their experience, inadequate instruction led to poor adherence because they were insecure and lacked confidence in whether they were properly doing the exercises at home or not. Although not all participants had negative experiences, all agreed that adequate exercise instruction was essential to gain confidence, perform the exercises efficiently, and to adhere to the exercise regimen.

Participant 7: *"I wanted to do exercises for at least two weeks at the centre, but she only gave me instructions on the first day, and she did not tell me if I was doing it correctly or not. In my house I was alone and I had pain, and I did not know if I was making a mistake with the exercises or if I was doing them too hard". (Female, 57 years)*

#### Giving reminders

Twelve participants experienced that when their care provider gave them specific reminders to exercise, it was useful to keep adherence to the exercise prescription. All of them specified that written or printed instructions were good reminders, assisting on adherence. Similarly, 7 participants reported that verbal instructions on how to insert the exercises into daily routine were also useful.

Participant 23: "*I did the exercises before going to bed because he (professional) told me I should do them at night time". (Female, 64 years)*

Participant 34: *"If they gave me a personal handout with explanations of the exercises and what I have to do each day, then seeing this personal programme reminded me and I got motivated to do the exercises". (Female, 48 years)*

#### Monitoring results and adherence to exercises

Twelve participants felt a strong motivation to perform the prescribed exercises at home when their care providers were regularly monitoring their adherence to the exercise programme, or their health status progress. Most of these participants mentioned that the monitoring was made by direct questions about their health status, progression, pain, or function.

Participant 27: *"When I went in the morning and he asked me, 'have you done the exercises,' or 'have you felt some improvement,' I got motivated to do the exercises" (Female, 48 years)*.

## Discussion

The results of this study demonstrate that some conducts of care provider and the contents of home-based exercise programmes were both important on participant's adherence to the programme. Care provider's style and home-based exercise programme conditions emerged as strong themes in our data.

### Home-based exercise programme conditions

Home-based exercise programmes is known to interfere with normal life and requires interruption of daily routine [29]. Our study is consistent with this statement, and evidences that participants presenting chronic neck or back pain decline more to adhere to prescribed home exercises when the home programme requires longer time for execution or includes exercises which are difficult to perform. Minimizing the interruption caused by exercising on daily routine may provide one solution to the poor adherence problem [29]. One solution would be limiting the number of exercises prescribed in each programme. Similarly, there is evidence suggesting that more than eight exercises in a programme play a negative influence on participant's adherence to prescribed exercises [38].

High levels of participant adherence have been closely related to their own perception of programme's benefits [30,39,40]. The influence of these benefits on participant's performance is an issue that supports the social cognitive theory [41]. Our study added empirical evidence that these benefits have a limited effect on participant's adherence until the point where participants have achieved their aimed outcomes. Therefore, it is recommendable that, when symptoms are absent, additional incentives should be provided in order to prevent recurrences [33]. For that reason, the consistent use of outcome measures, such as number of exercise repetition, endurance, or heart rate, would offer participants a sense of progress [23]. Knowing their own progress could offer them a sense of active control over their own health, which in turn, would be worthwhile when facing more important activities [42].

When adverse effects were perceived while performing the exercises, our participants naturally responded with a poor-adherence to the prescribed home programme. Patients suffering from knee osteoarthritis presented a similar response to exercise's adverse effects [27]. Similarly, pain increase has been suggested to contribute to low adherence rates, in accordance with a fear-avoidance model of inactivity [19]. Minimizing the pain and the fear associated to exercising must be a priority concern of care providers. In a review, Masters and Ogles [43] proposed that the use of entertainment while exercising can minimize sensation of discomfort and can improve participant's adherence. Our study suggests that a proper supervision during the exercise execution for the duration of session may be an additional element to reduce patients' insecurity and fear of exercising at home.

### Care provider's style

Our findings on the subject of how care provider's style is important for participant's adherence to home-based exercise programme confirm and extend previous findings [23]. The participants in this study recognized that a supervised instruction which includes proper feedback was important for their adherence. It is evidenced that exercises based only on written instructions are not often performed properly, and therefore lead to poorer outcomes than when compared with outcomes from exercises learned under the supervision of a care provider [44]. Nevertheless, there is also evidence that an interactive exercise mode combined with written instructions improves adherence to exercises of patients presenting back pain [45].

Written instructions or exercising during specific daily activities were usually used as reminders for our participants. The participants in our study often felt that these reminders were important. In a same way, the use of reminders has also been recommended by relevant studies, due to the fact that patients tend to forget exercising or have serious difficulties in fitting the exercises into their daily routine [46].

Our study demonstrated that what the care provider says when giving the exercise instruction is relevant for the patient's own decision-making process. In contrast, some author believes that offering information and justifying the efficacy of the treatment to patients, are not enough practice to manage successful treatment adherence in patients presenting chronic pain [47]. We considered that the efficacy of the information provided depends on whether or not it connects with the patients' beliefs and expectations. This is a central recommendation within the assessment of the bio-psycho-social model [48]. There is a wide literature on the subject of this model which is useful to strengthen treatment [49].

### Reflections

This study has been used to identify some of the connections between participant's experiences, their own perceptions, and their adherence behaviour. The results presented an insight into which factors the care provider shall consider in order to optimize participant's adherence when prescribing home-based exercises.

The strength of this study lies in the use of qualitative methodology to obtain a description of patients' experiences, the use of rigorous methods, and the use of an objective sampling frame and the selection of one heterogeneous sampling. This last issue suggests that results could be representative of the experience of patients with chronic neck or back pain.

Nevertheless, there were some weaknesses in our study. The study used a cross-sectional sample and interviews were limited to 1 interview per participant; this limits the ability to capture any changes over time. Participants who abandoned their treatment in the health care centre where excluded. The experiences of those participants who abandoned the prescribed regimen might help to lead conclusions in a different perspective of the adherence issue. Finally, participants were interviewed at 1, 2 or 3 months after their prescribed treatment in the health care centre and therefore results in relation to their adherence to the treatment period may be affect by a recall bias.

## Conclusions

Our study's subjects highlighted that adherence to treatment was poor when exercises were time consuming or when the programme interrupted the participant's daily routine. Additional issues which can difficult adherence were identified, such as time consumption, complexity and adverse effects of exercises, and some care provider's styles. Our results suggest that participant are most likely to adhere to home-based exercises when their care provider provides proper feedback and gives reminders during the supervised execution of exercises, and when the participants perceive the benefits of exercises on their pain status. Other important factors which can affect adherence to treatment are: the way in which the prescribed exercises are designed, the degree of difficulty of the exercises, and how the programme is delivered by the care provider. These findings provide additional information to health care providers, by showing which issues should be considered when delivering health care to patients presenting chronic neck or back pain.

## Competing interests

The authors declare that they have no competing interests.

## Authors' contributions

PER, FMM, JJGC and JMH participated in the design and analysis of the study. PER, FMM and JMH secured the funding and participated in the coordination of the project. PER, FJJS, SLOS, MEBA and RLV contributed to the data management. All authors helped to draft the manuscript, they read and approved the final manuscript.

## Appendix

### Focus group interview guide

1. Why did you go to the physiotherapist?

2. How did you feel about having neck or low back pain before starting with the physiotherapy treatment?

3. What have you been told about your chronic pain and its treatment?

4. Did you find it easy to adhere to the physiotherapist's instructions at the beginning of treatment? After your treatment started, was it easier to adhere to the instructions?

5. What kind of problems do you encounter to continue with the exercises when pain is no longer present?

6. Is there anything else you would like to say about your home-based exercise programme or your pain?

## Pre-publication history

The pre-publication history for this paper can be accessed here:

http://www.biomedcentral.com/1472-6963/10/60/prepub
